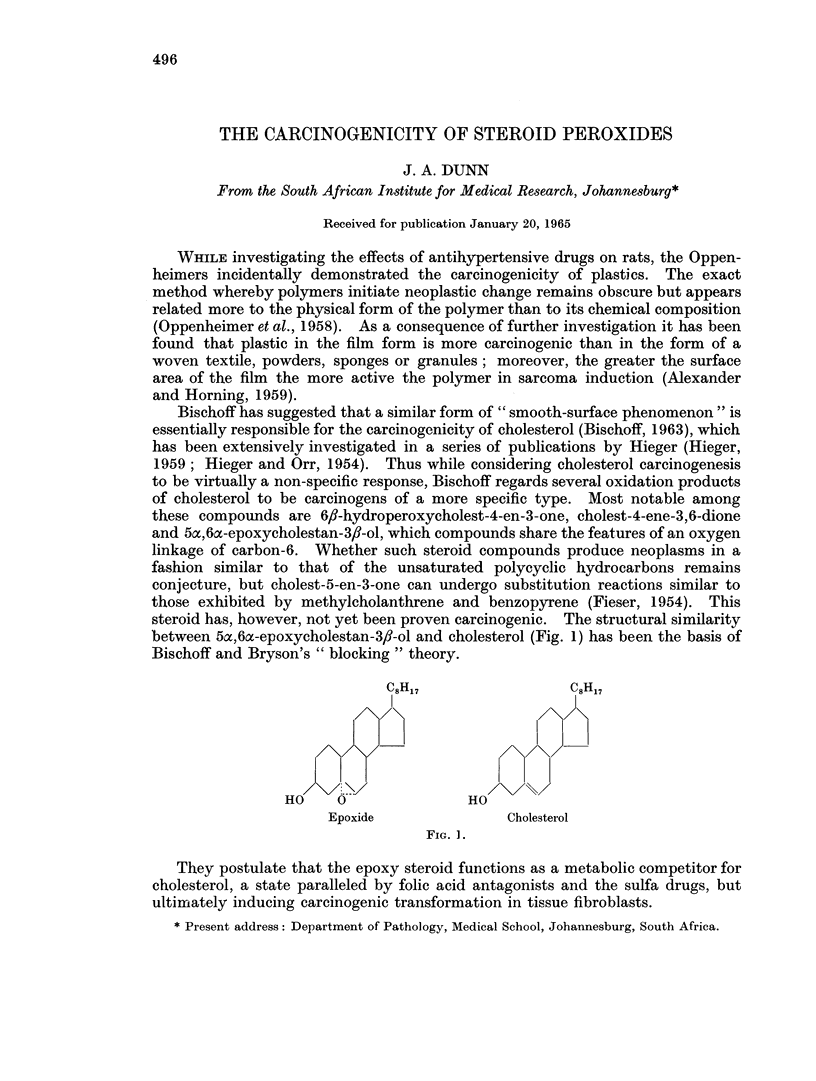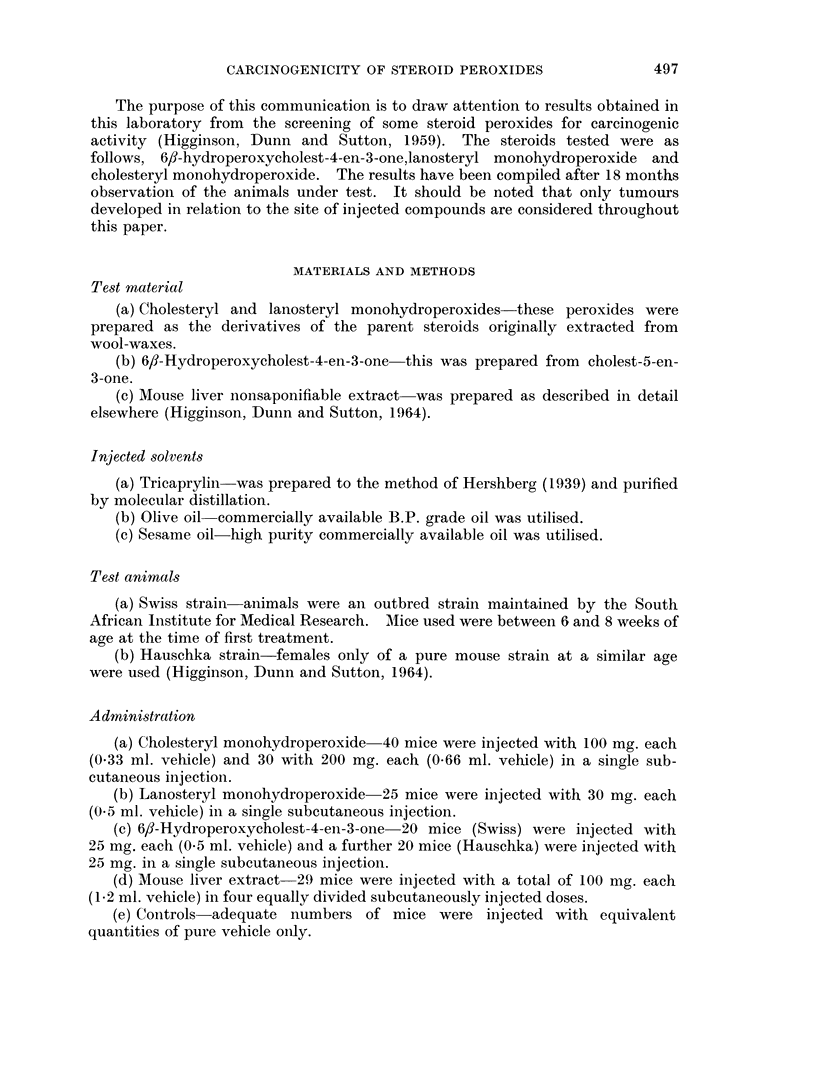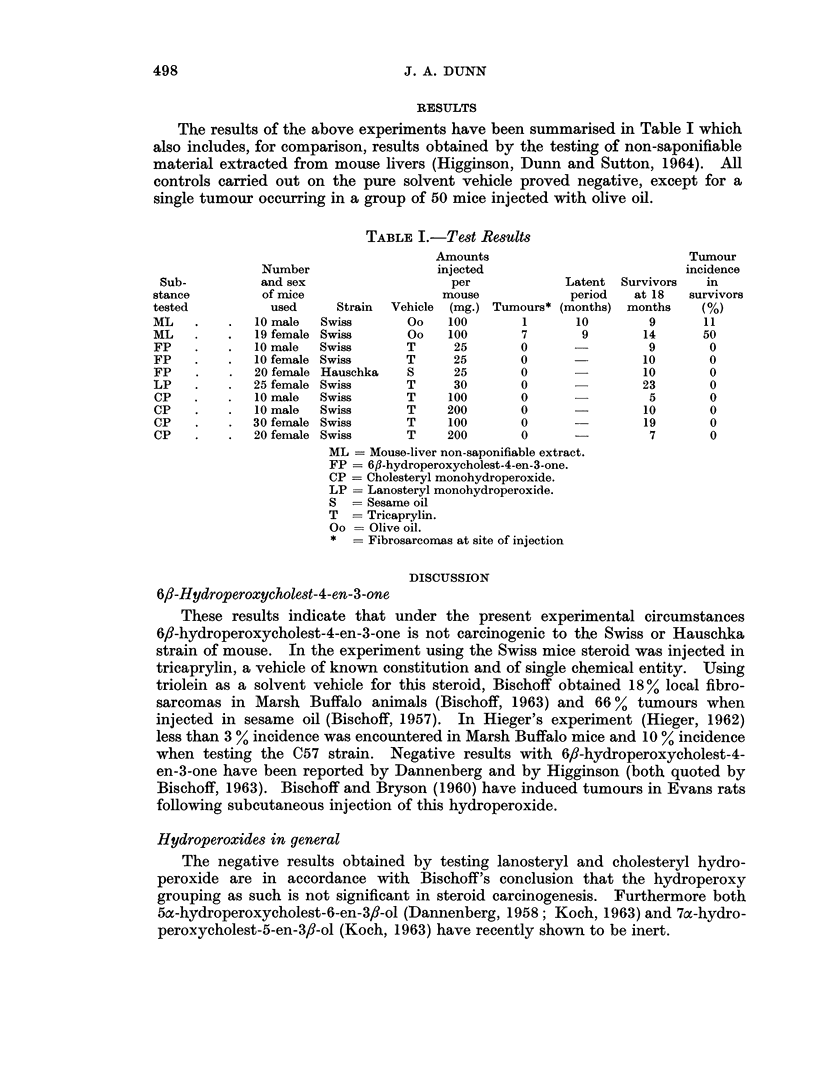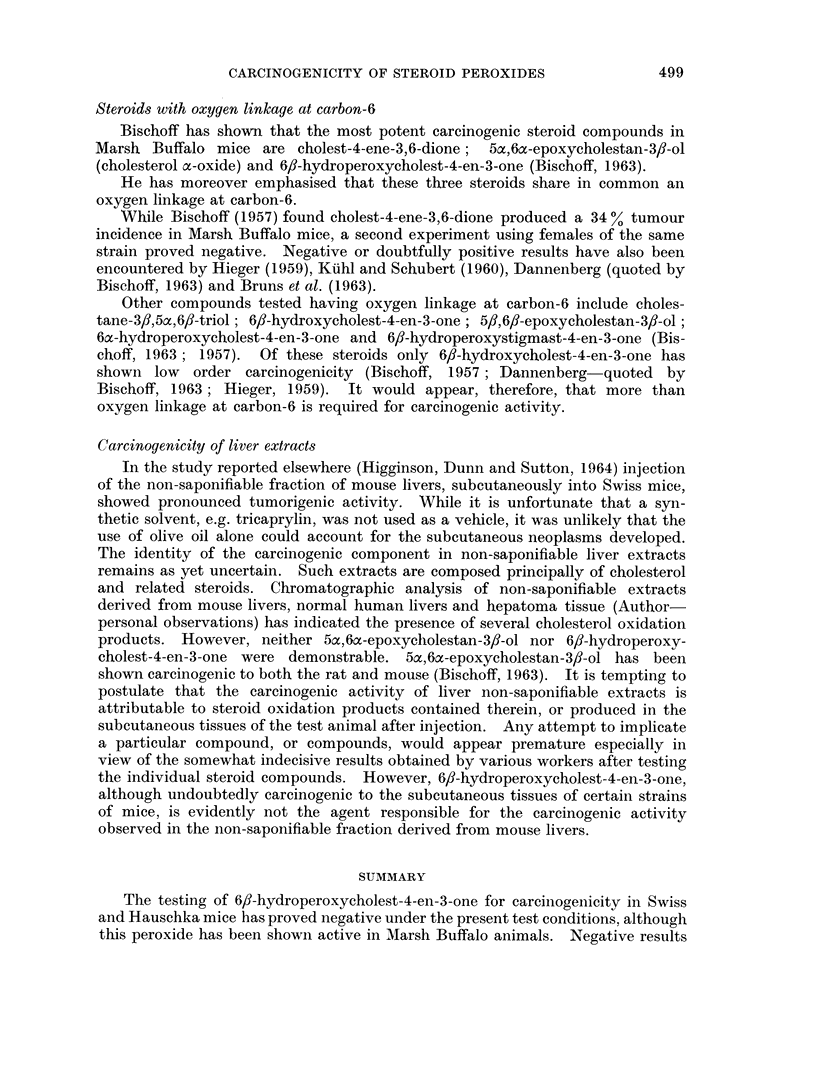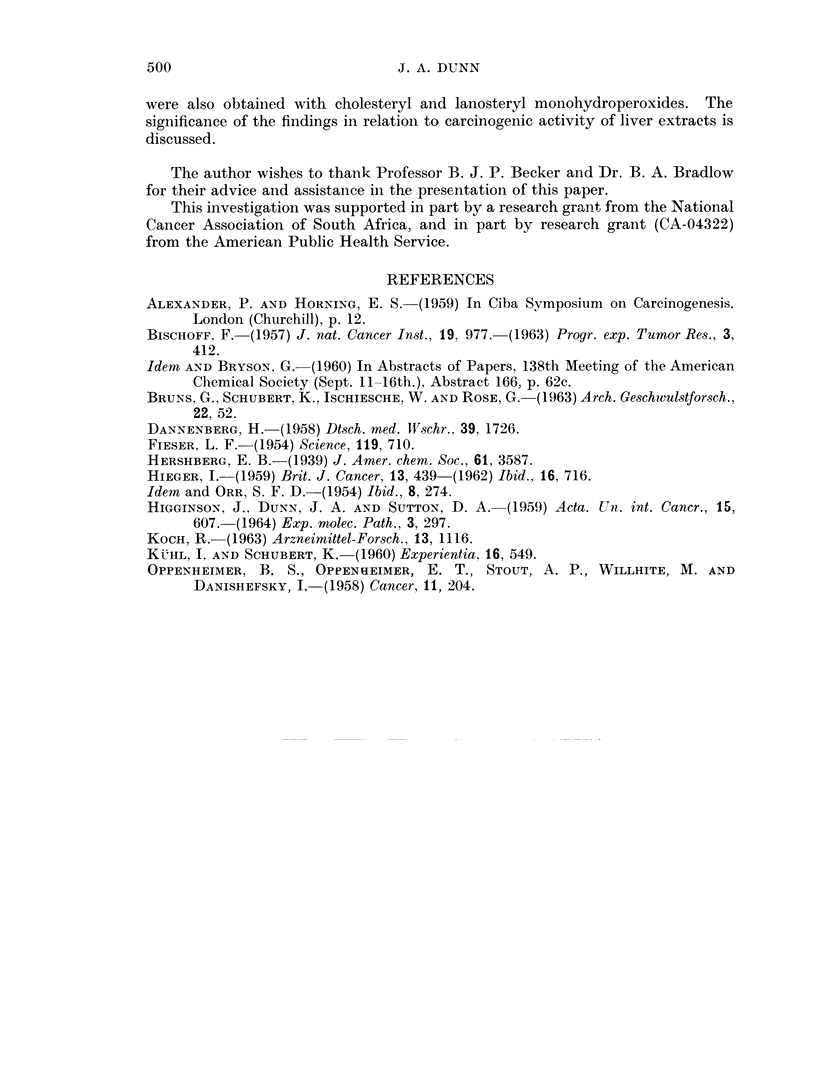# The carcinogenicity of steroid peroxides.

**DOI:** 10.1038/bjc.1965.59

**Published:** 1965-09

**Authors:** J. A. Dunn


### THE CARCINOGENICITY OF STEROID PEROXIDES

J. A. DUNN

*From the South African Institute for Medical Research, Johannesburg**

Received for publication January 20, 1965

WHILE investigating the effects of antihypertensive drugs on rats, the Oppenheimers incidentally demonstrated the carcinogenicity of plastics. The exact method whereby polymers initiate neoplastic change remains obscure but appears related more to the physical form of the polymer than to its chemical composition (Oppenheimer *et al.*, 1958). As a consequence of further investigation it has been found that plastic in the film form is more carcinogenic than in the form of a woven textile, powders, sponges or granules ; moreover, the greater the surface area of the film the more active the polymer in sarcoma induction (Alexander and Horning, 1959).

Bischoff has suggested that a similar form of "smooth-surface phenomenon" is essentially responsible for the carcinogenicity of cholesterol (Bischoff, 1963), which has been extensively investigated in a series of publications by Hieger (Hieger, 1959 ; Hieger and Orr, 1954). Thus while considering cholesterol carcinogenesis to be virtually a non-specific response, Bischoff regards several oxidation products of cholesterol to be carcinogens of a more specific type. Most notable among these compounds are 6β-hydroperoxycholest-4-en-3-one, cholest-4-ene-3,6-dione and 5α,6α-epoxycholestan-3β-ol, which compounds share the features of an oxygen linkage of carbon-6. Whether such steroid compounds produce neoplasms in a fashion similar to that of the unsaturated polycyclic hydrocarbons remains conjecture, but cholest-5-en-3-one can undergo substitution reactions similar to those exhibited by methylcholanthrene and benzopyrene (Fieser, 1954). This steroid has, however, not yet been proven carcinogenic. The structural similarity between 5α,6α-epoxycholestan-3β-ol and cholesterol (Fig. 1) has been the basis of Bischoff and Bryson's "blocking" theory.

FIG. 1.

They postulate that the epoxy steroid functions as a metabolic competitor for cholesterol, a state paralleled by folic acid antagonists and the sulfa drugs, but ultimately inducing carcinogenic transformation in tissue fibroblasts.

* Present address : Department of Pathology, Medical School, Johannesburg, South Africa.

The purpose of this communication is to draw attention to results obtained in this laboratory from the screening of some steroid peroxides for carcinogenic activity (Higginson, Dunn and Sutton, 1959). The steroids tested were as follows, 6$\beta$-hydroperoxycholest-4-en-3-one,lanosteryl monohydroperoxide and cholesteryl monohydroperoxide. The results have been compiled after 18 months observation of the animals under test. It should be noted that only tumours developed in relation to the site of injected compounds are considered throughout this paper.

### MATERIALS AND METHODS

*Test material*

(a) Cholesteryl and lanosteryl monohydroperoxides—these peroxides were prepared as the derivatives of the parent steroids originally extracted from wool-waxes.

(b) 6$\beta$-Hydroperoxycholest-4-en-3-one—this was prepared from cholest-5-en-3-one.

(c) Mouse liver nonsaponifiable extract—was prepared as described in detail elsewhere (Higginson, Dunn and Sutton, 1964).

*Injected solvents*

(a) Tricaprylin—was prepared to the method of Hershberg (1939) and purified by molecular distillation.

(b) Olive oil—commercially available B.P. grade oil was utilised.

(c) Sesame oil—high purity commercially available oil was utilised.

*Test animals*

(a) Swiss strain—animals were an outbred strain maintained by the South African Institute for Medical Research. Mice used were between 6 and 8 weeks of age at the time of first treatment.

(b) Hauschka strain—females only of a pure mouse strain at a similar age were used (Higginson, Dunn and Sutton, 1964).

*Administration*

(a) Cholesteryl monohydroperoxide—40 mice were injected with 100 mg. each (0·33 ml. vehicle) and 30 with 200 mg. each (0·66 ml. vehicle) in a single subcutaneous injection.

(b) Lanosteryl monohydroperoxide—25 mice were injected with 30 mg. each (0·5 ml. vehicle) in a single subcutaneous injection.

(c) 6$\beta$-Hydroperoxycholest-4-en-3-one—20 mice (Swiss) were injected with 25 mg. each (0·5 ml. vehicle) and a further 20 mice (Hauschka) were injected with 25 mg. in a single subcutaneous injection.

(d) Mouse liver extract—29 mice were injected with a total of 100 mg. each (1·2 ml. vehicle) in four equally divided subcutaneously injected doses.

(e) Controls—adequate numbers of mice were injected with equivalent quantities of pure vehicle only.

### RESULTS

The results of the above experiments have been summarised in Table I which also includes, for comparison, results obtained by the testing of non-saponifiable material extracted from mouse livers (Higginson, Dunn and Sutton, 1964). All controls carried out on the pure solvent vehicle proved negative, except for a single tumour occurring in a group of 50 mice injected with olive oil.

TABLE I.—*Test Results*

| Substance tested | Number and sex of mice used | Strain | Vehicle | Amounts injected per mouse (mg.) | Tumours* | Latent period (months) | Survivors at 18 months | Tumour incidence in survivors (%) |
|---|---|---|---|---|---|---|---|---|
| ML . . | 10 male | Swiss | Oo | 100 | 1 | 10 | 9 | 11 |
| ML . . | 19 female | Swiss | Oo | 100 | 7 | 9 | 14 | 50 |
| FP . . | 10 male | Swiss | T | 25 | 0 | — | 9 | 0 |
| FP . . | 10 female | Swiss | T | 25 | 0 | — | 10 | 0 |
| FP . . | 20 female | Hauschka | S | 25 | 0 | — | 10 | 0 |
| LP . . | 25 female | Swiss | T | 30 | 0 | — | 23 | 0 |
| CP . . | 10 male | Swiss | T | 100 | 0 | — | 5 | 0 |
| CP . . | 10 male | Swiss | T | 200 | 0 | — | 10 | 0 |
| CP . . | 30 female | Swiss | T | 100 | 0 | — | 19 | 0 |
| CP . . | 20 female | Swiss | T | 200 | 0 | — | 7 | 0 |

ML = Mouse-liver non-saponifiable extract.
FP = 6β-hydroperoxycholest-4-en-3-one.
CP = Cholesteryl monohydroperoxide.
LP = Lanosteryl monohydroperoxide.
S = Sesame oil
T = Tricaprylin.
Oo = Olive oil.
* = Fibrosarcomas at site of injection

### DISCUSSION

### *6β-Hydroperoxycholest-4-en-3-one*

These results indicate that under the present experimental circumstances 6β-hydroperoxycholest-4-en-3-one is not carcinogenic to the Swiss or Hauschka strain of mouse. In the experiment using the Swiss mice steroid was injected in tricaprylin, a vehicle of known constitution and of single chemical entity. Using triolein as a solvent vehicle for this steroid, Bischoff obtained 18% local fibrosarcomas in Marsh Buffalo animals (Bischoff, 1963) and 66% tumours when injected in sesame oil (Bischoff, 1957). In Hieger's experiment (Hieger, 1962) less than 3% incidence was encountered in Marsh Buffalo mice and 10% incidence when testing the C57 strain. Negative results with 6β-hydroperoxycholest-4-en-3-one have been reported by Dannenberg and by Higginson (both quoted by Bischoff, 1963). Bischoff and Bryson (1960) have induced tumours in Evans rats following subcutaneous injection of this hydroperoxide.

### *Hydroperoxides in general*

The negative results obtained by testing lanosteryl and cholesteryl hydroperoxide are in accordance with Bischoff's conclusion that the hydroperoxy grouping as such is not significant in steroid carcinogenesis. Furthermore both 5α-hydroperoxycholest-6-en-3β-ol (Dannenberg, 1958; Koch, 1963) and 7α-hydroperoxycholest-5-en-3β-ol (Koch, 1963) have recently shown to be inert.

*Steroids with oxygen linkage at carbon-6*

Bischoff has shown that the most potent carcinogenic steroid compounds in Marsh Buffalo mice are cholest-4-ene-3,6-dione ; 5$\alpha$,6$\alpha$-epoxycholestan-3$\beta$-ol (cholesterol $\alpha$-oxide) and 6$\beta$-hydroperoxycholest-4-en-3-one (Bischoff, 1963).

He has moreover emphasised that these three steroids share in common an oxygen linkage at carbon-6.

While Bischoff (1957) found cholest-4-ene-3,6-dione produced a 34% tumour incidence in Marsh Buffalo mice, a second experiment using females of the same strain proved negative. Negative or doubtfully positive results have also been encountered by Hieger (1959), Kühl and Schubert (1960), Dannenberg (quoted by Bischoff, 1963) and Bruns *et al.* (1963).

Other compounds tested having oxygen linkage at carbon-6 include cholestane-3$\beta$,5$\alpha$,6$\beta$-triol ; 6$\beta$-hydroxycholest-4-en-3-one ; 5$\beta$,6$\beta$-epoxycholestan-3$\beta$-ol ; 6$\alpha$-hydroperoxycholest-4-en-3-one and 6$\beta$-hydroperoxystigmast-4-en-3-one (Bischoff, 1963 ; 1957). Of these steroids only 6$\beta$-hydroxycholest-4-en-3-one has shown low order carcinogenicity (Bischoff, 1957 ; Dannenberg—quoted by Bischoff, 1963 ; Hieger, 1959). It would appear, therefore, that more than oxygen linkage at carbon-6 is required for carcinogenic activity.

*Carcinogenicity of liver extracts*

In the study reported elsewhere (Higginson, Dunn and Sutton, 1964) injection of the non-saponifiable fraction of mouse livers, subcutaneously into Swiss mice, showed pronounced tumorigenic activity. While it is unfortunate that a synthetic solvent, e.g. tricaprylin, was not used as a vehicle, it was unlikely that the use of olive oil alone could account for the subcutaneous neoplasms developed. The identity of the carcinogenic component in non-saponifiable liver extracts remains as yet uncertain. Such extracts are composed principally of cholesterol and related steroids. Chromatographic analysis of non-saponifiable extracts derived from mouse livers, normal human livers and hepatoma tissue (Author—personal observations) has indicated the presence of several cholesterol oxidation products. However, neither 5$\alpha$,6$\alpha$-epoxycholestan-3$\beta$-ol nor 6$\beta$-hydroperoxycholest-4-en-3-one were demonstrable. 5$\alpha$,6$\alpha$-epoxycholestan-3$\beta$-ol has been shown carcinogenic to both the rat and mouse (Bischoff, 1963). It is tempting to postulate that the carcinogenic activity of liver non-saponifiable extracts is attributable to steroid oxidation products contained therein, or produced in the subcutaneous tissues of the test animal after injection. Any attempt to implicate a particular compound, or compounds, would appear premature especially in view of the somewhat indecisive results obtained by various workers after testing the individual steroid compounds. However, 6$\beta$-hydroperoxycholest-4-en-3-one, although undoubtedly carcinogenic to the subcutaneous tissues of certain strains of mice, is evidently not the agent responsible for the carcinogenic activity observed in the non-saponifiable fraction derived from mouse livers.

### SUMMARY

The testing of 6$\beta$-hydroperoxycholest-4-en-3-one for carcinogenicity in Swiss and Hauschka mice has proved negative under the present test conditions, although this peroxide has been shown active in Marsh Buffalo animals. Negative results were also obtained with cholesteryl and lanosteryl monohydroperoxides. The significance of the findings in relation to carcinogenic activity of liver extracts is discussed.

The author wishes to thank Professor B. J. P. Becker and Dr. B. A. Bradlow for their advice and assistance in the presentation of this paper.

This investigation was supported in part by a research grant from the National Cancer Association of South Africa, and in part by research grant (CA-04322) from the American Public Health Service.

### REFERENCES

ALEXANDER, P. AND HORNING, E. S.—(1959) In Ciba Symposium on Carcinogenesis. London (Churchill), p. 12.

BISCHOFF, F.—(1957) *J. nat. Cancer Inst.*, **19**, 977.—(1963) *Progr. exp. Tumor Res.*, **3**, 412.

*Idem* AND BRYSON, G.—(1960) In Abstracts of Papers, 138th Meeting of the American Chemical Society (Sept. 11–16th.), Abstract 166, p. 62c.

BRUNS, G., SCHUBERT, K., ISCHIESCHE, W. AND ROSE, G.—(1963) *Arch. Geschwulstforsch.*, **22**, 52.

DANNENBERG, H.—(1958) *Dtsch. med. Wschr.*, **39**, 1726.

FIESER, L. F.—(1954) *Science*, **119**, 710.

HERSHBERG, E. B.—(1939) *J. Amer. chem. Soc.*, **61**, 3587.

HIEGER, I.—(1959) *Brit. J. Cancer*, **13**, 439—(1962) *Ibid.*, **16**, 716.

*Idem* and ORR, S. F. D.—(1954) *Ibid.*, **8**, 274.

HIGGINSON, J., DUNN, J. A. AND SUTTON, D. A.—(1959) *Acta. Un. int. Cancr.*, **15**, 607.—(1964) *Exp. molec. Path.*, **3**, 297.

KOCH, R.—(1963) *Arzneimittel-Forsch.*, **13**, 1116.

KÜHL, I. AND SCHUBERT, K.—(1960) *Experientia*, **16**, 549.

OPPENHEIMER, B. S., OPPENHEIMER, E. T., STOUT, A. P., WILLHITE, M. AND DANISHEFSKY, I.—(1958) *Cancer*, **11**, 204.